# A Challenge to the Colleges

**Published:** 1918-05-25

**Authors:** 


### The Hospital

The Workers' Newspaper of Administrative Medicine and Institutional Life, Administration, National Insurance and Health.

No. 1668, Vol. LXIV. SATURDAY, MAY 25, 1918. PRICE ONE PENNY

### A CHALLENGE TO THE COLLEGES.

The Royal Colleges of Physicians and Surgeons are, we imagine, widely regarded by the official world, and more or less indeed by the public generally, as the most exalted and most authoritative of the various organisations of the medical profession. They enjoy, without question, a considerable status, and they exercise a large influence on the views of various governing authorities. To the general body of the profession, however, they appear somewhat isolated and remote from the currents of actual affairs, and thus their proceedings are rarely the subject of anything like widespread professional interest. For the most part, indeed, they pursue a serene career undisturbed by the voices of the crowd. If we may judge from two recent utterances, this calm is likely to suffer a change. At all events, it happens that, presumably under the influence of the searching times in which we live, a critic of each of the two Colleges has stepped into the lists and has issued a challenge to battle.

The College of Surgeons finds its censor in no less a person than Sir Wm. Osler, and his occasion for speech is provided by the results of the last Primary Examination for the Fellowship. For this examination, it seems, thirty-four candidates presented themselves, and of these twenty-seven were rejected. The figures have a startling quality, but as a matter of fact they are hardly more emphatic than those exhibited on many previous occasions. Their immediate influence is to bring under review teachers, examiners, and students. In which of these groups lies the responsibility for so disastrous a record? Sir Wm. Osler exonerates each one of them. The teachers at the various hospitals, as is well known, conduct special courses of instruction for the examination. The examiners are chosen amongst the most distinguished Fellows of the College, and, as the examination is an honours test and only appeals to a select class, it follows that, with few exceptions, the candidates are the most ambitious and capable of the students. Yet, in spite of this combination, the latest examination record shows seven successes to twenty-seven failures! This situation Sir Wm. Osler pronounces an "intolerable" one, and all interested in medical education will, we feel sure, heartily agree with him. He blames no individual and no group of individuals, but he places the responsibility plainly and without qualification on "the rottenness of the present system." Obviously there must be some serious defect in an examination scheme which produces results such as those here under review, and it is impossible to regard with respect a test which displays these results. What happens at present is that, knowing the reputation of the examination, any candidate who proposes to attempt it feels compelled to take special classes, where he is "crammed" by teachers who are supposed to be experts in the peculiarities of the test. But these peculiarities, as the record shows, are not readily compassed, and they are applied, presumably under instructions, by examiners who reap their laurels in the slaughter of the great majority of the candidates. The unfortunate candidates themselves have only one consolation—namely, the knowledge that not a few men who now occupy distinguished positions suffered in their day a similar fate. The whole proceeding, in short, exhibits the examination system at its worst. It encourages, or even enforces, not thoughtful personal work, but "cramming." It is a test of memory, not of training and capacity. Its results are the sport of accident elevated into a system. And whilst it inflicts disappointment and humiliation on candidates the majority of whom are efficient and capable workers, success in meeting it means little more than an acquired dexterity to defeat deliberate, subtle and out-of-the-way issues. Sir William Osler does well to call for the entire abolition of this "rotten" scheme, and to claim that for it should be substituted a test arranged to discover what work the candidate has accomplished for himself, and not merely the degree of success with which his memory has been temporarily flooded by a knowledge of the work of others. The College is thus challenged by a voice that can hardly be ignored, and it will be of interest to note how it proposes to pick up the glove.

The critic of the College of Physicians appears anonymously in one of our medical contemporaries, and he cultivates a decidedly unsparing invective. Here again it is the mode of approach to the higher distinctions of the College that arms the criticism. The Fellowship in this instance is obtained, not by examination, but on the nomination of the College Council. And the charge is made that the nomination is made in an arbitrary and autocratic fashion, so that, while some of those who are eligible receive an early call, others not less worthy are postponed for years, or are even permanently ignored. Instances are given which certainly seem to bear out this allegation, and it must be allowed that no one seems to know what are the principles that govern election to the Fellowship. Of course, the reply may be that the College has a right to manage its own affairs in its own way. But equally the critic has a right to comment upon this management. Further, there is justice to individuals to be considered, and as the College has the privilege of conferring distinctions that are held to proclaim professional values there is a reasonable demand that it shall act consistently and apart from personal bias. The charge is now publicly made that it does not act on these principles, and whether a reply is or is not forthcoming the authorities must at least learn that their proceedings cannot hope to escape scrutiny. Medical policy generally will have to meet a stern test in the not distant future, and even the most exalted institutions may be called upon to justify their existence.

